# Susceptibility to SIV Infection After Adenoviral Vaccination in a Low Dose Rhesus Macaque Challenge Model

**DOI:** 10.20411/pai.v4i1.241

**Published:** 2019-01-29

**Authors:** Irene Bukh Brody, Roberto Calcedo, Mary J. Connell, Diane G. Carnathan, Martha Nason, Benton O. Lawson, Melon T. Nega, Surina Boyd, Qiuyue Qin, Thomas H. Vanderford, Jolaine M. Wilson, James M. Wilson, Guido Silvestri, Michael R. Betts

**Affiliations:** 1 Department of Microbiology; Perelman School of Medicine, University of Pennsylvania; Philadelphia, Pennsylvania; 2 Gene Therapy Program; Department of Medicine, Perelman School of Medicine, University of Pennsylvania; Philadelphia, Pennsylvania; 3 The Children's Hospital of Philadelphia Research Institute; Philadelphia, Pennsylvania; 4 Emory Vaccine Center; Yerkes National Primate Research Center, Emory University; Atlanta, Georgia; 5 Biostatistics Research Branch, National Institute of Allergy and Infectious Diseases, National Institutes of Health; Bethesda, Maryland; 6 University Laboratory Animal Resources; Perelman School of Medicine, University of Pennsylvania; Philadelphia, Pennsylvania

**Keywords:** vaccine, HIV, CD4, susceptibility

## Abstract

**Background:**

Vaccination with the Merck human adenovirus serotype-5 (HAdV-5) vectored HIV-1 subtype B gag/pol/nef vaccine was unexpectedly associated with enhanced susceptibility to HIV-1 infection in uncircumcised HAdV-5 seropositive men. It has been hypothesized that vaccination may have resulted in activated CD4+ T lymphocytes trafficking to mucosal sites thereby increasing targets for HIV infection. We have previously shown that AdV-vector vacci-nation in rhesus macaques resulted in an increase in the frequency of activated mucosal CD4+ T cells. However, whether this increase in activation is sufficient to increase susceptibility to HIV/SIV infection is unclear.

**Methods:**

To examine this scenario, we developed a preliminary, proof-of-concept vaccination-challenge model in order to examine vaccine-induced SIV susceptibility in rhesus macaques. Rhesus macaques (n = 10/group) were vaccinated with a simian AdV-7 (SAdV-7)-vector encoding an irrelevant insert (SARS spike) and challenged 5 weeks post-prime in an escalating dosing regimen starting with sub-infectious doses (1:10,000 or 2TCID_50_) of SIVmac251.

**Results:**

In contrast to our previous study, the SAdV-7 vaccine regimen did not induce detectable mucosal CD4+ T cell activation at the time points assessed in animals obtained from a different vendor and housed in a different facility. Within the power of the study, we did not observe significantly increased SIV acquisition in SAdV-7-vaccinated (5/10) versus placebo-vaccinated (3/10) macaques after repeated low-dose intra-rectal SIVmac251 challenge (*P* < 0.2).

**Conclusions:**

These results lay groundwork for future experiments to assess vaccine-induced SIV susceptibility in rhesus macaques. Further larger-scale studies are necessary to confirm the AdV-vector vaccination associated trend towards increased SIV/HIV acquisition and clarify associated mechanisms.

## INTRODUCTION

While enormous progress has been made towards an efficacious HIV vaccine, certain aspects of HIV biology have complicated the pursuit. The global HIV pandemic, currently infecting an estimated 34 million people, continues to be a public health concern due to the lack of clear correlates of immune protection, viral heterogeneity, and the difficulty of fully eradicating viral reservoirs. In line with this, 2 main avenues for vaccine design have been pursued, including induction of broadly neutralizing antibodies, as well as vector-based vaccines that generate sustained and robust HIV-specific T-cell responses [1, 2]. For the latter, recent large-scale clinical trials including Merck's STEP study [3], Phambili [4], and HVTN-505 [5] have all utilized recombinant replication-defective human adenovirus type-5 vectors (HAdV-5) to decrease HIV viral load or prevent HIV infection altogether. Unfortunately, all 3 trials failed to protect from HIV acquisition and were halted [5, 6]. In addition, participants in the STEP Trial revealed an enhanced susceptibility to HIV infection in HAdV-5 baseline seropositive, uncircumcised men [3, 7].

One hypothesis for the STEP study failure was that following vaccination of participants with pre-existing neutralizing antibodies (NAb) to HAdV-5, expansion and/or activation of memory CD4+ T cells that are already present in, and traffic to, the mucosa [8]—preferred target cells at the site of HIV transmission [9, 10]—may have increased HIV susceptibility. However, only peripheral blood samples were collected in these clinical trials, obviating the ability to directly assess potential mucosal-based mechanisms [3]. To address this experimentally, we previously examined CD4+ T-cell activation and AdV-specific CD4+ T-cell responses in the peripheral blood (PBMC) and rectal lamina propria (rLPL) after HAdV-5 and simian adenovirus type-7 (SAdV-7) vector vaccination using a rhesus macaque (*Macaca mulatta*) model. We found that the expression of activation markers on CD4+ T cells was heightened in the rLPL, but not the PBMC, after both HAdV-5 and SAdV-7 vaccination. Further, a statistically significant increase in the percentage of AdV-specific CD4+ T cells was found after AdV-vector vaccination in the rLPL [11]. Noting these differences between the peripheral blood and rectal mucosa, we initiated a proof-of-concept experiment to determine if AdV-based vector vaccination followed by SIV challenge during the activation window would increase SIV acquisition. Here, we assessed post-AdV vector vaccination induction of SIV susceptibility using an ultra-low dose SIV mucosal challenge. Unlike traditional vaccine studies where control animals are expected to become infected, we hypothesized that modeling potential vaccination-induced SIV susceptibility enhancement would necessitate an extremely low dose of virus challenge generally insufficient to infect the control animals. We therefore assessed susceptibility to dose-escalating intra-rectal challenges with SIVmac251 beginning at a minimally infectious dose (2TCID_50_), 5 weeks after a single immunization with an SAdV-7-based vector compared to a sham-vaccinated control. We did not find a significant increase in SIV acquisition with 5 SAdV-7-vaccinated versus 3 placebo-vaccinated macaques becoming SIV positive (*P* < 0.2). Together, these studies establish a model system with which to monitor potential vaccine-induced enhancement of SIV susceptibility in rhesus macaques.

## MATERIALS AND METHODS

### Adenovirus Vectors

Wild-type SAdV-7 was purchased from the ATCC (VR-201, originally isolated from rhesus monkey kidney cells). SAdV-7-based vectors containing an irrelevant transgene (SARS spike protein) were constructed as previously described [11, 12].

### Animals

Titration study: Fifteen healthy, SIV-uninfected Indian rhesus macaques were used in the SIV-mac251 titration study. All animals were housed at the Yerkes National Primate Research Center and in accordance with NIH guidelines. These studies were approved by the Emory University Institutional Animal Care and Use committee (IACUC).

Ad-vector vaccination challenge study: Twenty captive bred 5-year-old male Indian origin rhesus macaques were purchased from Covance Research Products (Alice, TX). All macaques were housed in the Children's Hospital of Philadelphia Colket Translational Research Building Large Animal Vivarium in accordance with the *Guide for the Care and Use of Laboratory Animals*, Public Health Service Policy, and Animal Welfare Act and Regulations in an AAALAC-accredited facility. All experiments were performed under protocols approved by The Children's Hospital of Philadelphia Institutional Animal Care and Use Committee.

### Titration Study Rectal Challenge with Infectious Simian Immunodeficiency Virus (SIVmac251)

All 15 macaques in the titration study were challenged intra-rectally each week with a low-dose of SIVmac251 (provided by Dr. Nancy Miller, DAIDS, NIAID, NIH, Bethesda, MD: see Supplementary Table 1). Challenges were performed with a 1 mL slip-tip syringe, containing 1 mL SIV-mac251 which was gently inserted ~4 cm into the rectum. A plunger was depressed to instill virus into the rectum. The macaque was then returned to its cage in a prone position. Challenge doses began at 4TCID_50_, which were repeated for 3 weeks, then increased to 20TCID_50_. This process was repeated for a total of 15 weeks, increasing by half a log every 3 weeks, until the animal became infected. The highest dose was 666TCID_50_. Macaques became infected at various doses over the course of the study, with > 1000 copies/mL considered infected. Animals were followed until day 42 post-infection.

### AdV-Vector Vaccination Study Rectal Challenge with SIVmac251

A 3 mL slip-tip syringe lightly coated with lubrication jelly and containing the specific SIVmac251 challenge dose was gently inserted into the rectum ~4cm, and the dose was slowly instilled. The animal remained in the prone position for at least 5 minutes after instillation of the virus. Animals were challenged weekly starting at week 5 post-vaccination, repeating the same dose for 3 weeks (once each week) and increased half a log every 3 weeks until the animal became infected. Challenge doses began at 1:10,000 (2TCID_50_), and the highest dose was 66.6TCID_50_.

### Immunizations

**Figure 1.**
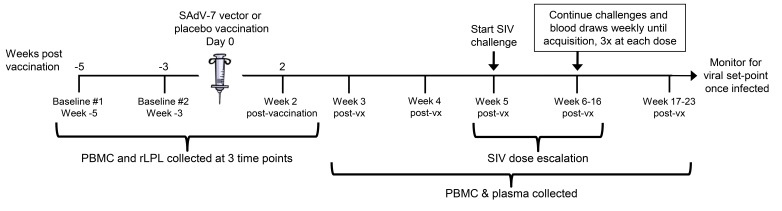
**Study timeline**. Overview of the 3 peripheral blood mononuclear cell (PBMC) and rectal lamina propria T lymphocyte (rLPL) collection time points interspersed around vaccination (day 0). Intra-rectal SIV challenges began at week 5 post-vaccination (post-vx) and continued until week 16.

Macaques in the vaccine group received a single, intramuscular (IM) injection of SAdV-7 at a dose of 1 × 10^11^ particles. Macaques in the placebo group similarly received a single IM injection of sterile saline as a control (see Figure 1 for the immunization schedule).

### Endoscopic Sampling of Macaque Rectum and Isolation of rLPLs

All rhesus macaques underwent fasting the evening before the procedure, with free access to water at all times. Then the animals were sedated, and biopsies were obtained utilizing an alligator-jaw style endoscopic biopsy pinch which was held free hand. Twenty biopsies were taken spaced far enough apart so as not to weaken the rectal wall. Biopsies were placed in RPMI medium and rLPLs isolated using collagenase type II as previously described [13].

### Blood Collection and Isolation of Peripheral Blood Mononuclear Cells

The PBMCs were isolated from whole blood collected in Vacutainer CPT Cell Preparation Tubes with Sodium Heparin (Ref 362753, Becton, Dickinson and Company, Franklin Lakes, NJ) following the protocol recommended by the vendor. Briefly, tubes were initially centrifuged at 1700 RCF at ambient temperature to isolate the mononuclear cells. After centrifugation, the mononu-clear cells were resuspended into the plasma by tube inversion and all contents above the gel were pipetted into a separate 15 mL conical tube. Then the PBMCs were centrifuged at 300 RCF and washed in PBS following the protocol.

### AdV Neutralizing Antibody Assay

Anti-SAdV-7 neutralizing antibody titers in serum samples were measured by assessing the ability of serum to inhibit transduction of the corresponding reporter vector SAdV-7LacZ into HEK 293 cells as previously described [11]. The NAb titer was reported as the highest reciprocal serum dilution that inhibited AdV.CMV.LacZ transduction (β-gal expression) by 50%, compared with the serum control. The limit of detection of the assay is 5.

### Antibody Reagents

Antibodies used for surface staining included anti-CD14 Qdot 655, anti-CD20 Qdot 655, anti-CD4 PeCy5.5, anti-CD8 Qdot605 (Invitrogen; Carlsbad, California), anti-CD14 BV650, anti-CD20 BV650 (Biolegend; San Diego, California), anti-CD28 ECD (Beckman Coulter; Fullerton, CA), anti-CD25 APC-Cy7, anti-CD95 PE-Cy5, and anti-HLA-DR APC (BD Pharmingen; San Diego, California). Antibodies used for intracellular staining included anti-interleukin-2 (IL-2) Alexa700 (Biolegend), anti-interferon-gamma (IFNγ) Alexa 700 (Invitrogen), anti-tumor necrosis factor-alpha (TNFα) Alexa 700, anti-CD3 Pac Blue, anti-CD69 PE, and anti-Ki67 FITC (BD Pharmingen).

### Determination of Plasma Viral RNA (Viral Load)

SIVmac251 loads were measured in plasma samples by real-time PCR as previously described [14].

### Cell Processing and Stimulation

Rectal biopsies were processed within 6 hours of being collected. Rhesus macaque PBMCs were cryopreserved in fetal bovine serum (FBS; ICS Hyclone, Logan, Utah) containing 10% dimethyl sulfoxide (DMSO; Fisher Scientific, Pittsburgh, Pennsylvania) and stored in liquid nitrogen until use. After washing fresh rLPLs or thawed PBMCs once in RPMI (Mediatech Inc; Manassas, Virginia), both PBMCs and rLPLs were resuspended in complete medium (RPMI supplemented with 10% FBS, 1% L-glutamine [Mediatech Inc] and 1% penicillin-streptomycin [Lonza; Walkers-ville, Maryland], sterile filtered) at a concentration of 1-2 × 10^6^ cells/mL medium in FACS tubes. Cells were split into 3 stimulation conditions, at a volume of 1 mL each, with no stimulation, or 1 µL Staphylococcus Enterotoxin B (SEB) at a concentration of 1 mg/mL (Sigma-Aldrich; St. Louis, Missouri) as a positive control, or 1 × 10^10^ particles/mL of the SAdV-7 vector. Cells were stimulated overnight at 37°C, 5% CO_2_.

### FACS Staining Assay

Stimulation tubes were removed from the incubator in the morning to add monensin (0.7 µg/ml final concentration; BD Biosciences) and brefeldin A (1 µg/mL final concentration; Sigma-Aldrich; St. Louis, Missouri) and incubated for an additional 6 hours. Cells were then washed once with PBS and stained for viability with Aqua amine-reactive dye (Invitrogen) for 10 minutes in the dark at room temperature. A mixture of antibodies used for staining surface markers was added to the cells and kept at room temperature for 20 minutes. Cells were washed with PBS containing 1% bovine serum albumin (BSA, Fisher Scientific) and 0.1% sodium azide (Fisher Scientific) and permeabilized for an additional 20 minutes at room temperature using the Cytofix/Cytoperm kit (BD Pharmingen). Next, cells were washed in Perm/Wash buffer (BD Pharmingen), and a mixture of antibodies used for staining intracellular markers was added to the cells and incubated in the dark for 1 hour at room temperature. Cells were again washed with Perm/Wash buffer and fixed with PBS containing 1% paraformaldehyde (Sigma-Aldrich). Fixed cells were stored in the dark at 4°C until collection.

### Flow Cytometric Analysis

For each sample, between 3 × 10^5^ and 1 × 10^6^ total events were acquired on a modified flow cytometer (LSRII; BD Immunocytometry Systems; San Jose, CA) equipped to detect up to 18 fluorescent parameters. Antibody capture beads (BD Biosciences) were used to prepare individual compensation tubes for each antibody used in the experiment. Data analysis was performed using FlowJo version 9.0.1 (TreeStar, Ashland, Oregon). Percentage expression is shown after background subtraction, where values are calculated as the difference between cells that were stimulated with SAdV-7 vector overnight minus those that were left unstimulated. AdV-specific percentages are reported as the population of CD4+ memory T cells which express IL-2, IFNγ, and/or TNFα within each compartment. To separate naive cells from memory, effector memory, and effector T cells, we stained with fluorochrome-conjugated antibodies for CD28 and CD95, where CD28^+^ CD95-CD4+ T cells indicated the naive subset, and all other cells were grouped as memory cells. Naive CD4+ T cells were gated using FlowJo for each macaque at all time points in both compartments.

### Figures

Prism software, version 5.0 (Graphpad; La Jolla, California) was used to create the figures.

### Statistical Analysis

This study was designed to have sufficient power (80%) to detect a significant result (*P* < 0.05) with group differences in SIV acquisition if 0/10 of the animals in the placebo group and 5/10 in the vaccine group became infected, or 1/10 in the placebo group and 7/10 in the vaccine group became infected. The groups were compared with respect to the probability of infection as a function of challenge dose using a logistic regression model. In order to account for the structure of the data, *P* values were computed based on a permutation null distribution. The permutation null distribution was created by randomly permuting the group labels between animals 1000 times and recomputing the statistic after each permutation; the observed statistic was then compared to this permutation null distribution.

## RESULTS

### SIVmac251 Low-Dose Titration

We recently described the development and use of the simian adenovirus type 7 (SAdV-7) vector in order to model natural adenovirus immunological responses in macaques compared to using a human AdV vector [11, 12]. In order to assess whether the vaccine-induced mucosal CD4+ T-cell activation we previously observed induced a heightened state of SIV infection susceptibility, we first developed an ultra-low dose challenge system, wherein control animals were less likely to be infected compared to the vaccinated animals. Therefore, we began by performing a low-dose intra-rectal titration of our SIVmac251 stock in 15 macaques. The lowest concentration was a 1:5000 (4TCID_50_) dilution that, in previous titration studies involving only a few macaques, appeared to produce little or no SIV infection. Two of 15 macaques were infected at the lowest 1:5000 dose (Supplementary Table 1). As a result, we started our vaccination-challenge study at 1:10,000 (2TCID_50_), in order minimize the chance of infection in the control group and highlight any potential enhancement effect in the vaccine group.

### Simian Adenovirus Type 7 Immunization and Challenge Study Design

Twenty Indian-origin rhesus macaques were stratified into vaccine and placebo groups based on group distribution of baseline cytokine and activation marker expression, as well as baseline SAdV-7 neutralizing antibody (NAb) titers (Supplementary Table 2). SAdV-7 NAb titers ranged from < 5 (undetectable) to 640 in 1 macaque and did not correlate with SIV acquisition by the end of the study. We vaccinated 10 macaques intramuscularly with 1 × 10^11^ viral particles (VP) of an E1-deleted replication-defective SAdV-7 vector diluted in sterile saline (see Materials and Methods for construct description). As a control, we vaccinated 10 macaques with sterile saline. PBMCs and rLPLs were obtained at 2 baseline time points prior to immunization (week -5 and week -3) and week 2 post-vaccination (Figure 1). Once rectal pinch biopsies were collected at week 2 post-vaccination, macaques were rested for 3 weeks to allow healing of the rectal mucosa tissue before challenge. Based on our previous study, AdV-induced rLPL CD4+ T-cell activation peaked at 5 weeks and decreased nearly to baseline by 16 weeks post-SAdV-7 vaccination. For this reason, we chose to structure the timeline such that the peak window of activation overlapped with the SIV challenge window. Macaques were intra-rectally challenged weekly with SIVmac251 starting at week 5, beginning with a 1:10,000 (2TCID_50_) dose dilution. Each challenge dose was administered 3 times before increasing the dilution half a log, until SIV acquisition, as indicated by weekly plasma viral load assays. SIV challenges continued until week 16 (1:300 or 66.6TCID_50_), at which point 12 macaques had not acquired SIV. Macaques were monitored for viral set-point once infected.

### Immunologic Assessment of SAdV-7 Vectored Vaccine Induced Responses

Previously, we found that SAdV-7 vector vaccination preferentially induced strong AdV-specific T-cell responses, heightened CD4+ T-cell activation, and decreased frequency of naive CD4+ T cells in the rectal lamina propria of vaccinated macaques (37). As such, we first sought to confirm that the SAdV-7 vector used here similarly induced these changes within the rectal lamina propria following immunization.

**Figure 2.**
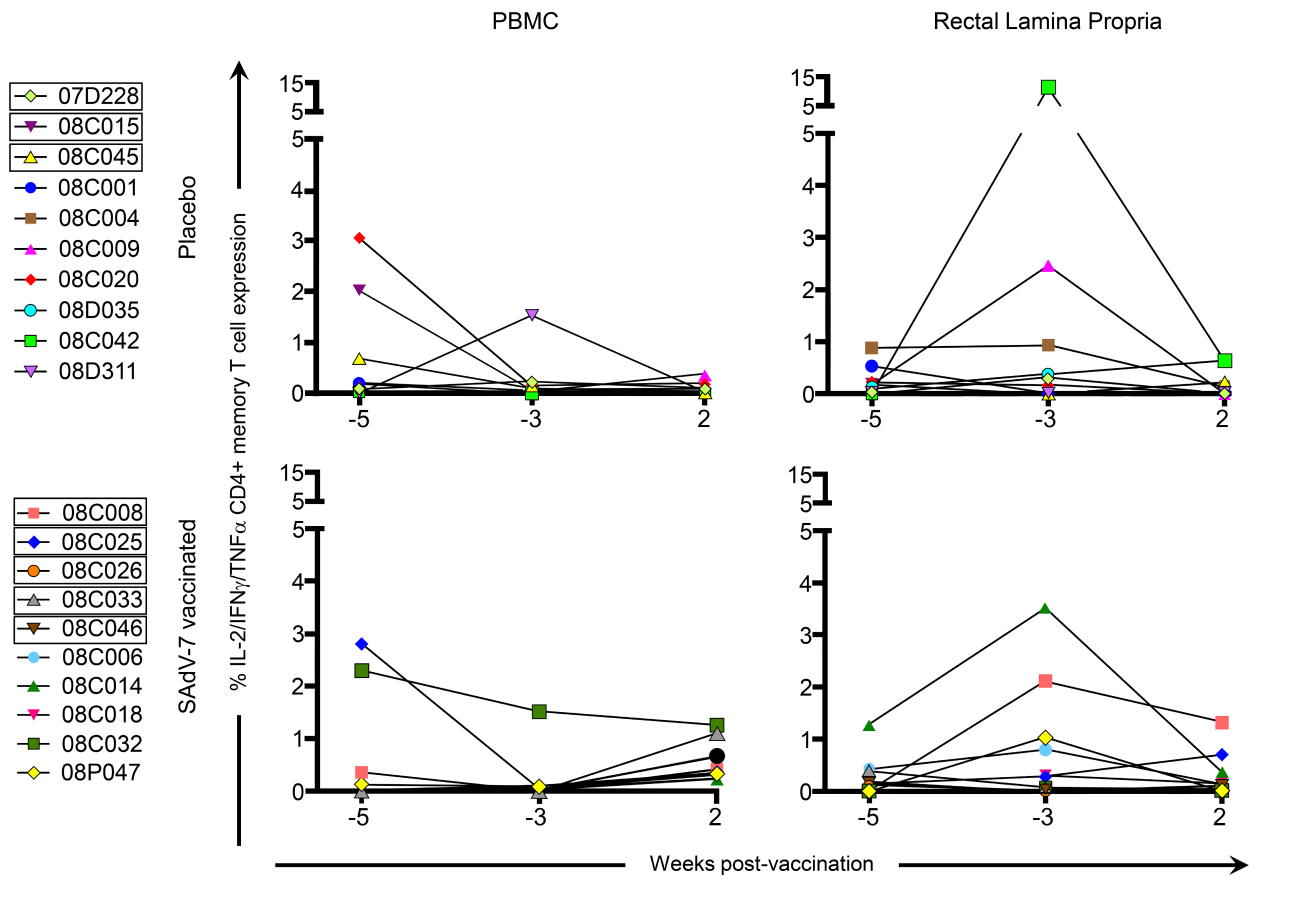
**CD4+ memory AdV-specific T-cell cytokine responses in the PBMC and rLPL**. Cytokine responses (IL-2, IFNγ, TNFα) were combined for SAdV-7-stimulated (overnight) CD4+ memory T cells and baseline subtracted from unstimulated cells. Values are shown for both baseline time points (weeks -5 and -3) and week 2 post-vaccination. Boxes around macaque numbers in the legend indicate animals that became SIV+ during the challenge.

**Supplementary Figure 1.**
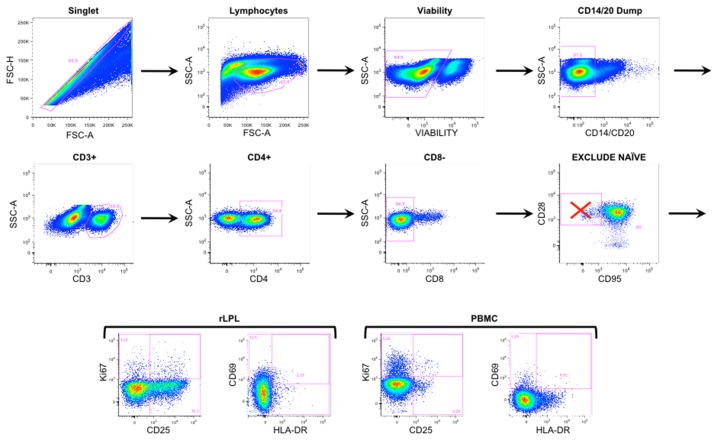
**Representative flow cytometry gating.** Rectal LPL and PBMCs were gated on single cells, including live lymphocytes and excluding CD14/20 cells. Within the CD3+ pool, CD4+/CD8-T cells further excluded naive (CD28+/CD95-) T cells. Plots on the top 2 rows were obtained from rLPL at baseline week -5. Four bottom panels represent activation marker expression in rLPL (left) and PBMC (right).

Baseline AdV-specific CD4+ T cells were detectable for all macaques in at least 1 compartment (Figure 2, representative data shown in Supplementary Figure 1), with 7/10 SAdV-7-vaccinated and 9/10 placebo-vaccinated macaques showing responses in the peripheral blood, and 10/10 SAdV-7-vaccinated and 9/10 placebo-vaccinated macaques showing responses in the rectal mucosa (response > 0.05% was considered positive). However, while SAdV-7 vaccination resulted in a greater average percentage of AdV-specific CD4+ T cells in both PBMC (0.55%) and rLPL (0.28%) at week 2 relative to placebo vaccination (PBMC 0.09%, rLPL 0.16%), this increase was not statistically significant.

**Figure 3.**
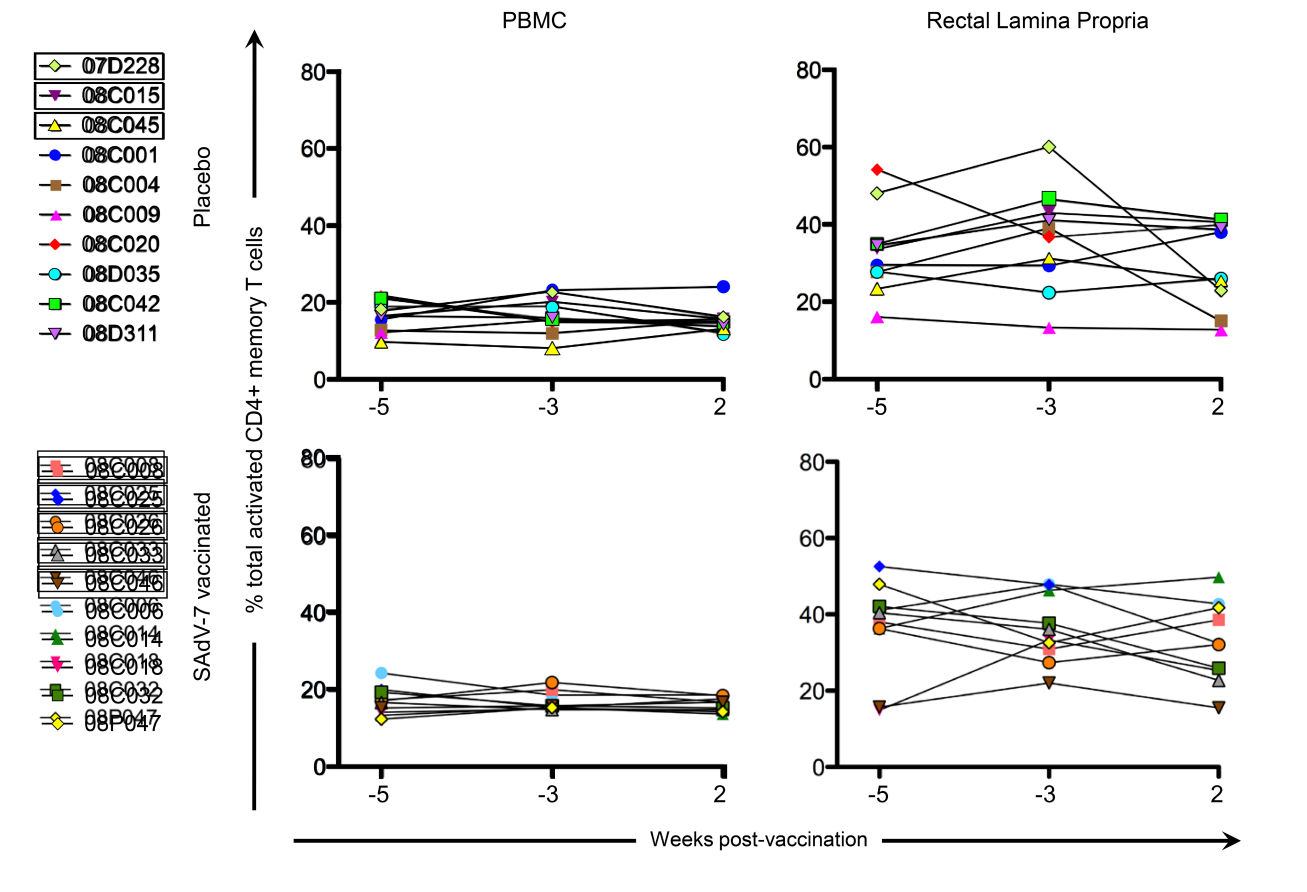
**Percentage of total activated CD4+ memory T cells without stimulation**. Values are shown for both baseline time points (weeks -5 and -3) and week 2 post-vaccination in the peripheral blood (PBMC) and rectal lamina propria. Activation values for each macaque represent the summed percentages of any CD4+ memory T cell that expresses at least 1 (or more) of the 3 activation markers measured in this study (HLA-DR, CD25, and/or Ki67) within that time point. Boxes around macaque numbers in the legend indicate animals that became SIV+ during the challenge.

We next assessed CD4+ T-cell activation prior to immunization (weeks -5 and -3), and week 2 post-vaccination (Figure 3) to determine whether vaccination with this SAdV-7 vector induced heightened mucosal CD4+ T-cell activation. However, unlike our previous study, we found no significant changes in the percentage of total activated CD4+ memory T cells (representing the summed percentages of any CD4+ memory T cell that expressed at least 1, or more, of the 3 activation markers including HLA-DR, CD25, and/or Ki67) before and after vaccination. As expected, the rectal lamina propria had, on average, consistently higher expression of activation markers compared to PBMCs. Moreover, the percentage total CD4+ T-cell activation marker expression was comparable between the placebo and vaccine groups within each compartment at both baseline and week 2 post-vaccination.

**Figure 4.**
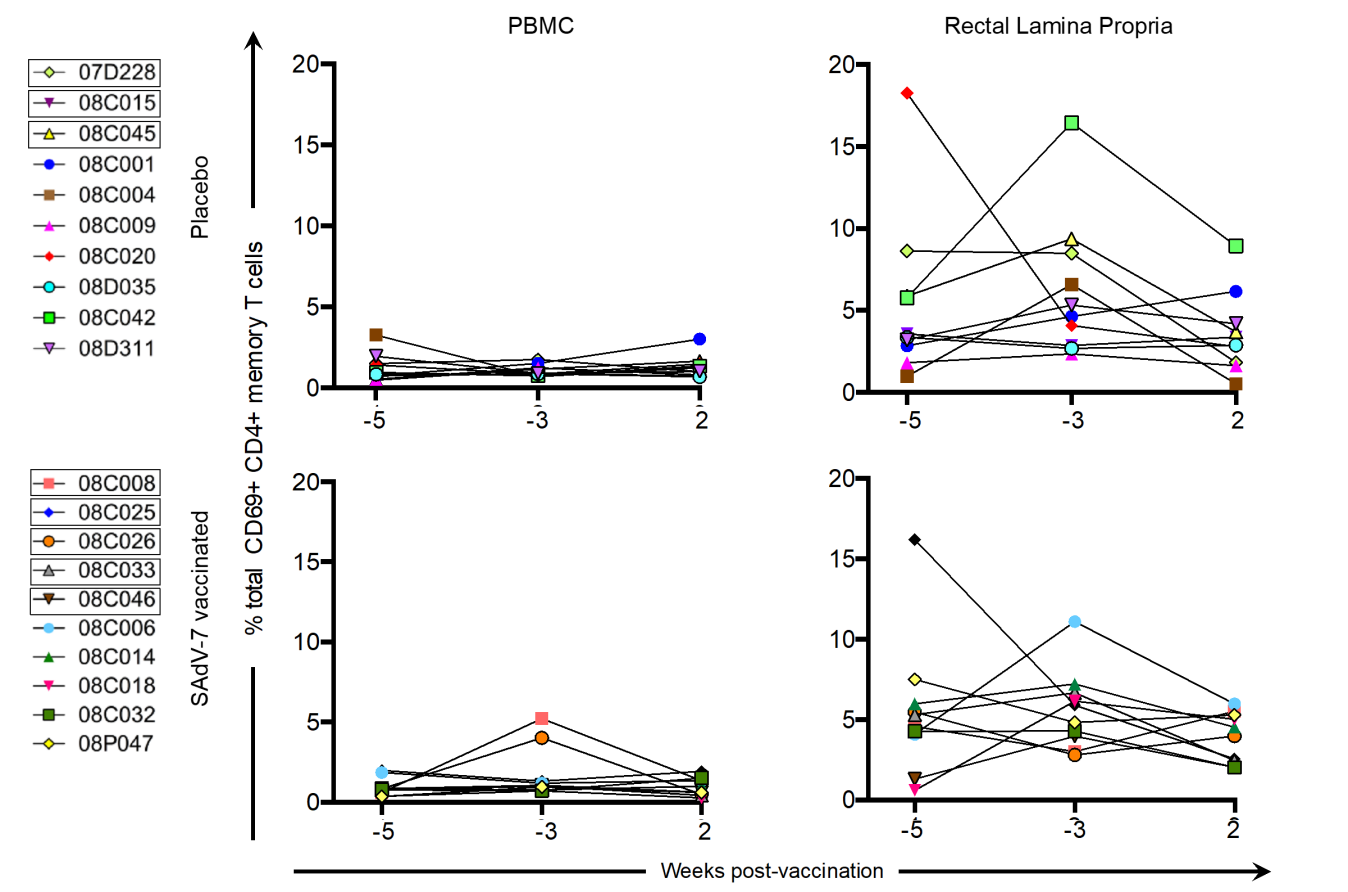
**Percentage of total CD69+ CD4+ memory T cells without stimulation.** Values are shown for both baseline time points (weeks -5 and -3) and week 2 post-vaccination in the peripheral blood (PBMC) and rectal lamina propria. Points for each macaque represent the percentage of CD69+ CD4+ memory T cell expression (resident T cells) within that time point. Boxes around macaque numbers in the legend indicate animals that became SIV+ during the challenge.

We also assessed CD69+ CD4+ T-cell expression at the same time points prior to and post-vaccination (Figure 4) to investigate whether SAdV-7 vector vaccination altered tissue-resident memory (T_RM_) CD4+ T-cell populations. Similar to the levels of total CD4+ T-cell activation, the rectal lamina propria had higher average CD69+ CD4+ memory T-cell expression than PBMCs. Furthermore, no significant difference was seen between baseline (week -5 and -3) time points and week 2 post-vaccination in either compartment or with either group indicating CD4+ T_RM_ populations were not altered initially by vaccination.

**Figure 5.**
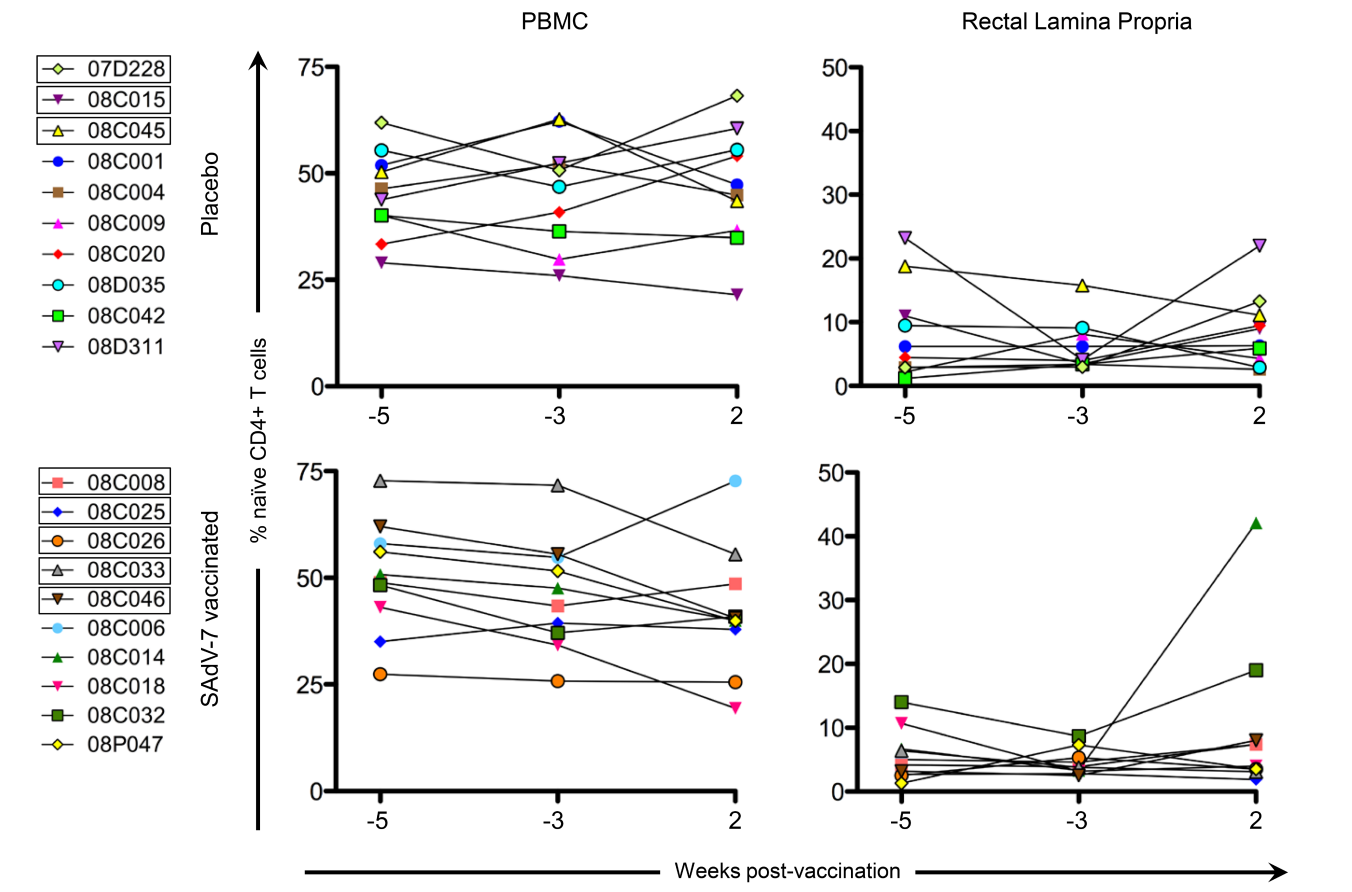
**Percentage of naive CD4+ T cells at each time point**. Naive cells are CD28+/CD95-by flow cytometry (a representative plot can be found in Supp. Fig. 1). All cells were SAdV-7-stimulated overnight before fluorochrome staining. Values are shown for both baseline time points (weeks -5 and -3) and week 2 post-vaccination in the peripheral blood (PBMC) and rectal lamina propria. Boxes around macaque numbers in the legend indicate animals that became SIV+ during the challenge.

Finally, we measured naive CD4+ T cells (Figure 5), defined as CD28+ CD95-, in the peripheral blood and rectal lamina propria for cells stimulated with SAdV-7 overnight at all 3 time points. As expected, the average relative percentage of naive CD4+ T cells was much higher in PBMCs (baseline placebo 45.62% vs baseline SAdV-7-vaccinated 48.18%; week 2 placebo 46.7% vs week 2 SAdV-7-vaccinated 42.09%) than in the rLPLs (baseline placebo 7.15% vs baseline SAdV-7-vaccinated 5.11%; week 2 placebo 8.69% vs week 2 SAdV-7-vaccinated 10%). Overall, the values did not fluctuate significantly before and after vaccination in any group, except for SAdV-7-vaccinated macaques #08C014 displaying an unusually high naive CD4+ T-cell percentage at week 2 post-vaccination. Taken together, these results indicate that the SAdV-7 vector performed quite differently in this group of macaques compared to the animals in our previous study.

### Trend Towards SIV Acquisition After SAdV-7 Vector Vaccination Relative to Placebo

**Figure 6.**
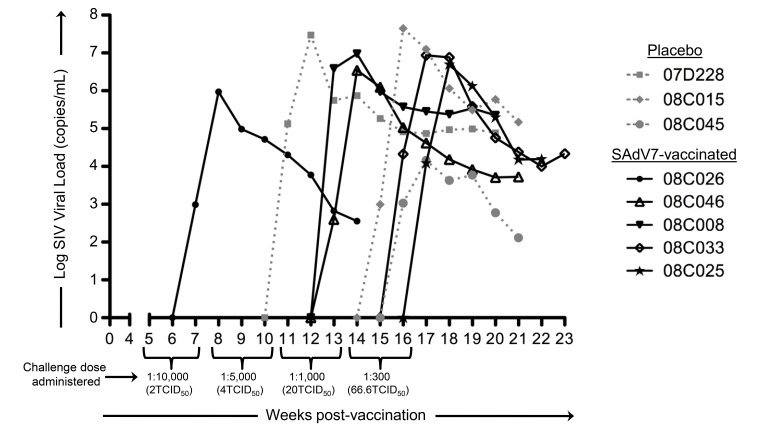
**Viral loads in SIV-infected macaques after dose-escalating intra-rectal challenges**. Three placebo-vaccinated macaques (gray dotted lines) and 5 SAdV-7-vaccinated macaques (black solid lines) acquired SIV as a result of weekly SIV challenges (*P* < 0.2). Challenge doses were administered 3x/dose before increasing to the next dose to all 20 macaques. SIV challenges began at 5 weeks post-vaccination and continued until 16 weeks post-vaccination.

Starting at week 5 after either SAdV-7 vector or placebo vaccination, we began weekly intra-rectal low-dose SIV challenges at 1:10,000 (2TCID_50_) for all 20 macaques (Supplementary Table 3). Although we did not see evidence of increased AdV-specific or activated CD4+ T cells at week 2 post-vaccination, the week 5 challenge time point was chosen based on the peak activation levels observed in our previous study, which peaked at week 5 and subsided by week 16 (37). As shown in Figure 6, 5 SAdV-7-vaccinated macaques and 3 placebo-vaccinated macaques became infected throughout the challenge course, but this was not statistically significant (*P* < 0.2) within the power of the study. The time of SIV acquisition did not occur with any preference towards the vaccination group. Peak viral loads for SAdV-7-vaccinated macaques ranged from 9.34 × 10^5^ (#08C026) to 9.53 × 10^6^ (#08C008) copies/mL and in placebo-vaccinated macaques ranged from 1.45 × 10^4^ (#08C045) to 2.96 × 10^7^ (#07D228) copies/mL. Although macaques were not always followed through to final viral load set-point, final viral loads prior to euthanasia were not consistently higher or lower for either the vaccination group (range of set-points for SAdV-7-vaccinated animals was 3.57 × 10^2^ [#08C026] to 2.24 × 10^5^ [#08C008] and for placebo-vaccinated animals was 1.3 × 10^2^ [#08C045] to 1.49 × 10^5^ [#08C015]). Finally, we found no association between pre-challenge activation levels, AdV-specific CD4+ T-cell frequency, or pre-vaccination AdV titers (data not shown) and infection status or infection rate within either the vaccine or placebo groups.

## DISCUSSION

Numerous follow-up studies have aimed to understand the mechanisms of HAdV-5-based vector vaccination clinical trial failures, without any definitive answer. Some of these studies have attempted to elucidate the outcome of the STEP Study through examination of the peripheral blood only [15-17], leading to subsequent reports citing the value of both peripheral blood and gastrointestinal mucosa analyses. Our study was designed to clarify if increased frequencies of AdV-specific CD4+ T cells and CD4+ T-cell activation levels in the gut mucosa after AdV-based vector vaccination that were evident in our previous study would lead to increased SIV susceptibility after challenge. We did not find a significant increase in SIV acquisition after intra-rectal low-dose challenges in SAdV-7-vaccinated macaques (5/10) compared to placebo-vaccinated macaques (3/10) (*P* < 0.2). With a small sample size of 10 macaques per group, and only 1 post-vacci-nation sample collection time point, we believe that additional exploration is necessary to determine whether AdV-vectored vaccination results in increased SIV infection susceptibility.

The clear effects of AdV-vectored vaccination that we were able to show in our previous study, which displayed significant increases after vaccination relative to baseline in both AdV-specific CD4+ T cells and activated CD4+ T cells in the rectal mucosa, were not evident in in this study. Although we did verify that all the SAdV-7 vector-vaccinated macaques (except #08C006) did seroconvert by week 2 post-vaccination, macaques, like humans, show substantial variation, and this can be seen in immunological responses. One caveat of this study is that the genetic diversity found (including MHC class I alleles, TRIM5α) can make smaller studies, such as these, difficult to perform. Additionally, these animals came from a different source and with different housing conditions than our previous study, both of which may be a factor in the baseline and post-vacci-nation responses. We saw higher baseline levels of AdV-specific CD4+ memory T-cell responses, albeit insignificantly, than previously. Also, at week 2 post-vaccination, none of the macaques had AdV-specific CD4+ T-cell responses above ~2%, whereas in our previous study, 5 macaques had elevated AdV-specific CD4+ T-cell responses in the rLPL. AdV-specific CD4+ memory T cells reached statistically significant increases only starting at week 5 post-vaccination in our last study, so it is possible that if we were able to wait longer to assess these responses, we may have seen changes here as well. Further, the percentage of total activated CD4+ memory T cells at baseline in the rectal mucosa was substantially higher in the current study (~15%-50% vs ~10%-30% previously), although levels of activation in the peripheral blood were similar. It is unclear what the cause of this large difference is, but it is possible that macaques in this study were exposed to more antigens at baseline or had a substantially different gut microbiome leading to differential antigen-induced CD4+ T-cell activation. In the ideal situation, animals could be screened for mucosal T-cell activation and Ad-sp nAb titers prior to study inclusion; however, the cost of acquiring enough animals for a properly powered study makes this untenable. Alternatively, animals could be repeatedly vaccinated with Ad vectors to induce higher Ad-sp nAb titers. However, this would still not reflect natural life-long exposure to infectious Adenovirus in humans.

There are a few main factors to consider that influenced the design of this study. The primary focus of our study design was to synchronize the SIV challenges with the peak of SAdV-7 vector vaccination-induced mucosal CD4+ T-cell activation, which (from our first study) occurred at approximately 2-5 weeks post-vaccination and gradually subsided by week 16. We hypothesized that exposure to SIV during this time period would lead to increased SIV acquisition in the SAdV-7 vector-vaccinated group relative to the placebo group. However, we were only able to obtain a single post-vaccination week 2 rectal biopsy to assess vaccine-induced effects in order to provide a long enough time for the rectal mucosa to heal prior to SIV challenge at week 5. Based on our previous data where several macaques showed peaks of activation after week 2 [11], we recognized that immunological dynamics might be slightly different in this group of macaques and we could miss sampling the rectal mucosa activation peak. Finally, we were limited by relatively small groups (20 macaques split evenly into vaccine and placebo recipients), which limited our statistical power. While we saw a trend towards increased SIV acquisition in the SAdV-7 vector-vaccinated group, this was unfortunately not enough to reach statistical significance. The conclusion from the STEP Study that the Merck HAdV-5 HIV-1 subtype B gag/pol/nef vaccine may have increased HIV acquisition was based on 49/914 male vaccine recipients versus 33/922 male placebo recipients acquiring HIV [3]. Therefore, this is a difference of 16 infections in 1,836 men (0.87%) between vaccine and placebo groups—a relatively marginal increase in HIV acquisition, but significant due to the large size of the study population.

Unifying the HIV vaccine community towards an appropriate nonhuman primate model to illustrate immunological and virological outcomes from clinical efficacy trials using AdV-based vectors and other vector-based HIV vaccines will be critically important as the field progresses. The failure of the STEP Trial, Phambili, and HVTN-505 were disappointing, but were fairly consistent with previous SIV vaccine studies using HAdV-5 vectors [18-20]. Although the mechanism for the failure of the STEP Study has yet to (and may never) be resolved, the developmental path of any HIV vaccine should thoughtfully employ the nonhuman primate model in such a way to directly assess potential vaccine enhancement effects at relevant challenge sites. The studies we have described here provide an initial strategy upon which future studies can build to improve our ability to properly assess potential detrimental effects of suboptimal HIV vaccine strategies.

## SUPPLEMENTARY MATERIALS

**Supplementary Table 1.** **SIVmac251 low-dose intra-rectal titration**. Fifteen rhesus macaques (RMs) were SIV challenged intra-rectally weekly and viral loads were monitored weekly until SIV acquisition. Challenge doses began at 1:5000 (4TCID_50_) and were repeated 3 times before increasing by half a log every 3 weeks, until SIV infection occurred. All 15 macaques became SIV+ by the 14th challenge at 1:30 dilution.

Challenge Dose Causing Infection	TCID_50_ Equivalent	# of RMs Infected	Challenge #
1:5000	4TCID_50_	2	3
1:1000	20TCID_50_	2	4
1:1000	20TCID_50_	2	5
1:1000	20TCID_50_	1	6
1:300	66.6TCID_50_	3	8
1:300	66.6TCID_50_	1	9
1:100	200TCID_50_	1	10
1:100	200TCID_50_	1	12
1:30	666TCID_50_	1	13

**Supplementary Table 2.** **SAdV-7 baseline neutralizing antibody (NAb) titers**. Titers are reported for placebo-vaccinated and SAdV-7-vaccinated macaques, the assay is described in the Materials & Methods. (^*^) indicates that the macaque acquired SIV before the study completion.

Monkey ID	SAdV-7 baseline NAb titer	SAdV-7 week 2 NAb titer
**Placebo group**		
07D228*	80	40
08C015*	20	20
08C045*	5	< 20
08C001	40	20
08C004	< 5	< 20
08C009	< 5	< 20
08C020	< 5	< 20
08C042	80	40
08D035	5	< 20
08D311	640	320
**SAdV-7 vaccinated**		
08C008*	80	1280
08C025*	< 5	80
08C026*	20	80
08C033*	80	1280
08C046*	20	640
08C006	40	20
08C014	80	1280
08C018	5	320
08C032	40	160
08P047	5	80

^*^ Indicates SIV+ by the end of the study

**Supplementary Table 3.** **SIV viral loads in rhesus macaques**. Numerical presentation of data shown in Figure 5. Three placebo-vaccinated and 5 SAdV-7-vaccinated macaques became SIV+ and were sacrificed when viral loads started to reach set-point. The dose causing infection is administered 1 week prior to first detectable viral load. Undet = undetectable, assay sensitivity is 60 copies/mL.
